# The Public Health Payoff of “No Smoking Allowed”: Quantifying Decreases in SHS Exposure

**Published:** 2006-06

**Authors:** Angela Spivey

Secondhand smoke (SHS), the cigarette smoke involuntarily inhaled by a nonsmoker, was recognized as a serious health problem as early as 1972. Studies have shown that SHS causes lung cancer and leads to other adverse effects, including lower respiratory tract infections, bronchitis, pneumonia, fluid in the middle ear, and sudden infant death syndrome. Fortunately, a new report by researchers at the CDC shows that over the last 14 years, SHS exposure has decreased substantially—by an average of 70% in people over the age of 4, regardless of sex, age, or race **[*EHP* 114:853–858; Pirkle et al.]**.

The researchers analyzed data from the National Health and Nutrition Examination Survey (NHANES), conducted by the CDC’s National Center for Health Statistics, which collected data on the U.S. population during four distinct time periods from 1988 through 2002. Over the 14-year span, 29,849 nonsmoking participants completed a home interview followed by a physical exam.

SHS exposure was measured by testing blood samples from each participant for serum levels of cotinine, the primary byproduct formed when the body metabolizes nicotine. Serum cotinine levels indicate SHS exposure that occurred in the past few days. Nonsmokers exposed to typical levels of SHS usually have serum cotinine concentrations of less than 1 nanogram per milliliter (ng/mL), while active smokers generally have concentrations greater than 15 ng/mL. For this study, participants with serum cotinine concentrations of less than 10 ng/mL were considered nonsmokers (the relatively high cutoff accommodates heavy exposure to SHS).

During the first time period studied, 1988 through 1991, 65% of nonsmokers had serum cotinine concentrations greater than 0.1 ng/mL. On that basis, Healthy People 2010 (a DHHS initiative) had earlier established an objective of reducing that percentage to 45% by the year 2010. The current study results suggest that this goal had already been met by 2000. The authors say the decrease is most likely due to restrictions on smoking at work and in other public places, since adult smoking itself did not decrease dramatically in the 1990s.

Although public health efforts have been successful overall, the results suggest that further efforts should focus on two groups that showed relatively higher levels of risk of SHS exposure—children and blacks. In the most recent time period studied, 2001 through 2002, the median exposure level for children aged 4 to 11 was almost twice that of adults: 0.067 ng/mL, compared to 0.035 ng/mL. The median exposure level for blacks was even higher, at 0.135 ng/mL, compared to 0.034 ng/mL for whites.

Previous studies have shown that blacks have consistently higher serum cotinine concentrations per cigarette smoked than do whites. But other studies have found higher SHS levels among blacks even after accounting for differences in metabolism. The consistently higher serum cotinine concentrations for black nonsmokers in the current study appear to reflect higher SHS exposure, the authors state, though metabolism differences may have influenced the numbers somewhat.

To focus on these at-risk groups, further public health efforts are needed to discourage smoking where children are present, inside homes, and inside cars, the authors conclude.

## Figures and Tables

**Figure:**
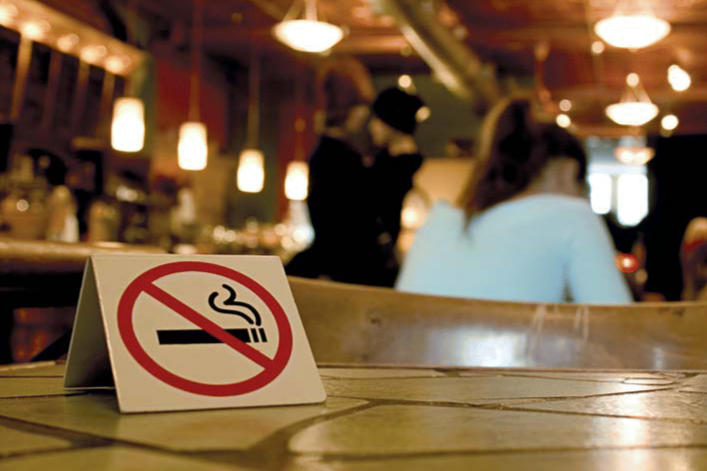
Signs of progress A new study shows that antismoking campaigns have greatly reduced exposures to SHS, but additional measures are still needed to protect at-risk groups such as children and blacks.

